# Everolimus induces Met inactivation by disrupting the FKBP12/Met complex

**DOI:** 10.18632/oncotarget.9484

**Published:** 2016-05-19

**Authors:** Lucia Raimondo, Valentina D'Amato, Alberto Servetto, Roberta Rosa, Roberta Marciano, Luigi Formisano, Concetta Di Mauro, Roberta Clara Orsini, Priscilla Cascetta, Paola Ciciola, Ana Paula De Maio, Maria Flavia Di Renzo, Sandro Cosconati, Agostino Bruno, Antonio Randazzo, Filomena Napolitano, Nunzia Montuori, Bianca Maria Veneziani, Sabino De Placido, Roberto Bianco

**Affiliations:** ^1^ Department of Clinical Medicine and Surgery, University of Naples “Federico II”, Naples, Italy; ^2^ Department of Oncology, University of Turin, Candiolo Cancer Institute - FPO IRCCS, Turin, Italy; ^3^ DiSTABiF, Second University of Naples, Caserta, Italy; ^4^ Department of Pharmacy, University of Naples “Federico II”, Naples, Italy; ^5^ Department of Translational Medical Sciences, University of Naples “Federico II”, Naples, Italy; ^6^ Department of Molecular Medicine and Medical Biotechnologies, University of Naples “Federico II”, Naples, Italy

**Keywords:** everolimus, Met, FKBP12, everolimus resistance

## Abstract

Inhibition of the mechanistic target of rapamycin (mTOR) is a promising treatment strategy for several cancer types. Rapamycin derivatives such as everolimus are allosteric mTOR inhibitors acting through interaction with the intracellular immunophilin FKBP12, a prolyl isomerase with different cellular functions. Although mTOR inhibitors have significantly improved survival of different cancer patients, resistance and lack of predictive factors of response remain unsolved issues. To elucidate the mechanisms of resistance to everolimus, we evaluated Met activation in everolimus-sensitive/resistant human cancer cells, *in vitro* and *in vivo*. Biochemical and computational analyses were performed. Everolimus-resistant cells were xenografted into mice (10/group) and studied for their response to everolimus and Met inhibitors. The statistical significance of the *in vitro* results was evaluated by Student's *t* test.

Everolimus reduced Met phosphorylation in everolimus-sensitive cells. This event was mediated by the formation of a Met-FKBP12 complex, which in turn is disrupted by everolimus. Aberrant Met activation in everolimus-resistant cells and overexpression of wild-type/mutant Met caused everolimus resistance. Pharmacological inhibition and RNA silencing of Met are effective in condition of everolimus resistance (*P*<0.01). In mice xenografted with everolimus-resistant cells, the combination of everolimus with the Met inhibitor PHA665752 reduced tumor growth and induced a statistically significant survival advantage (combination *vs* control *P*=0.0005).

FKBP12 binding is required for full Met activation and everolimus can inhibit Met. Persistent Met activation might sustain everolimus resistance. These results identify a novel everolimus mechanism of action and suggest the development of clinical strategies based on Met inhibitors in everolimus-resistant cancers.

## INTRODUCTION

Everolimus (RAD001) is an allosteric inhibitor of mechanistic target of rapamycin complex 1 (mTORC1) that is effective in the treatment of different cancer types: advanced breast cancer, renal cell carcinoma, and neuroendocrine tumors of pancreatic origin [1–4]. It exerts its effect by binding to the intracellular immunophilin FK506/rapamycin binding protein 12 (FKBP12). The resulting inhibitory complex binds with high affinity to mTORC1 affecting downstream effectors and ultimately inhibiting tumor cell proliferation [5]. FKBP12 is the prototype FKBP; it contains only one FK506/rapamycin-binding domain, which consists of 108 amino acids. FKBP12 constitutively associates with IP_3_ (inositol triphosphate) [6], binds Ras in a palmitoylation-dependent fashion promoting retrograde trafficking of Ras, and also binds and regulates the activity of cellular membrane receptors endowed with kinase activity such as TGFbeta and EGFR [7–9].

Everolimus have gained FDA approval for the treatment of metastatic renal cell carcinoma, for hormone receptor-positive, epidermal growth factor receptor 2 (HER2)-negative breast cancer and for pancreatic neuroendocrine tumors [10, 4, 11]. Clinical trials are currently ongoing on several tumor types, including non small cell lung cancer, gastic, ovarian, thyroid, pancreatic carcinomas [12]. Data from early-phase studies indicate that only a subset of patients derive significant clinical benefit from treatment with mTOR inhibitors [13]. The molecular basis of sensitivity and resistance to everolimus is largely unknown. Among the molecular mechanisms of resistance to mTOR inhibitors, different studies have described mutations in FKBP-12 or mTOR, PI3K/AKT or ERK/MAPK pathway activation via upregulation of receptor tyrosine kinases (RTKs), altered expression levels of eIF4E and 4E-BP1, modulation of apoptotic regulators, oxidative stress, enhanced angiogenesis, stimulation of autophagy [14].

Met is a transmembrane RTK for the hepatocyte growth factor (HGF), whose ligand-induced activation promotes such biological activities as cell proliferation, cell invasion and protection from apoptosis. The HGF/Met axis drives resistance to targeted therapies in several ways, and preclinical data suggest that combinatorial therapies with Met inhibitors is a promising anticancer approach [15].

In this study, we asked whether Met activation could affect everolimus sensitivity, and if so, whether pharmacological inhibition of Met could be a strategy in patients with everolimus resistance.

## RESULTS

### Everolimus inhibits Met phosphorylation in various human cancer cell lines

**Figure 1:**
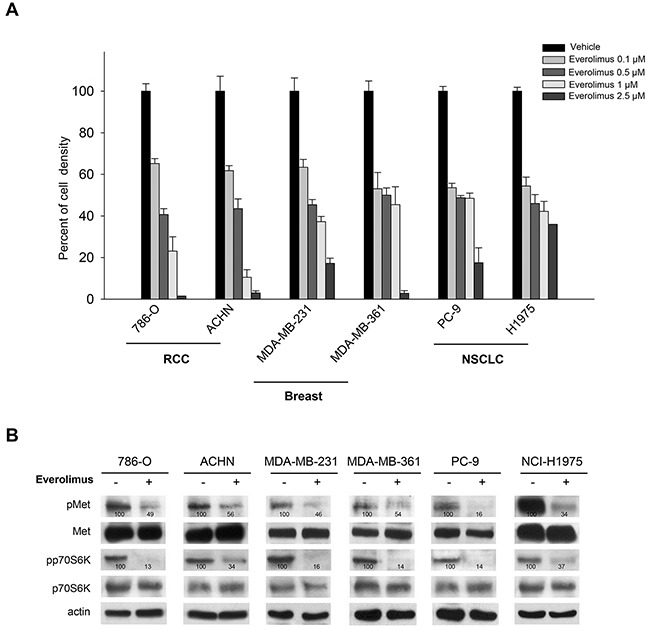
Everolimus is effective and inhibits Met phosphorylation in different human cancer cell lines **A.** Percent of cell density of human renal cell carcinoma (786-O, ACHN), breast (MDA-MB-231, MDA-MB-361), non small cell lung cancer (PC-9, NCI-H1975) cells treated for 72 hours with everolimus (0.1 − 2.5 μM), as measured by MTT assay. Data represent the mean (±SD) of three independent experiments, each performed in triplicate. Bars, SDs. **B.** Western blot analysis of protein expression in 786-O, ACHN, MDA-MB-231, MDA-MB-361, PC-9, NCI-H1975 cells treated for 24 hours with everolimus (0.5 μM). The relative optical density of phospho-protein levels normalized to total protein levels is shown.

We selected human cancer cell lines sensitive to everolimus: renal (786-O and ACHN), breast (MDA-MB-231 and MDA-MB-361), and lung (PC-9 and NCI-H1975) cancer cells. We first verified the *in vitro* sensitivity of these cell lines to everolimus in cell survival assays, and found that all cell lines were highly sensitive to everolimus. The concentration of everolimus causing 50% reduction of cell density was ≤ 0.5 μM (*P* < 0.0001) (Figure 1A, Supplementary Table S1).

Since rapalogs have been reported to induce a negative feedback on some RTKs [16], we investigated the activation status of different RTKs upon everolimus treatment (data not shown) and surprisingly, we found an alteration of Met RTK. Particularly, in renal, breast and lung cell lines, decreased p70S6K phosphorylation paralleled inhibition of Met phosphorylation (Figure 1B).

### Met phosphorylation is not reduced after mTOR inhibition

To evaluate if the phospho-Met reduction occurring upon everolimus treatment could depend from direct inhibition of the Met TK, we performed an *in vitro* kinase assay comparing the effect of everolimus with that of the Met inhibitor PHA665752 on a number of Met TK variants, both wild-type (wt) and mutants. As shown in Table 1, everolimus did not inhibit any of the isolated Met TK variants (IC_50_ > 10 μM). Conversely, PHA665752 inhibited Met TK variants albeit to different degrees, the effect being greatest against Met wt (IC_50_ < 100 nM). This suggested that the phospho-Met reduction could depend on mTOR inhibition. To test this hypothesis, we evaluated the activation/phosphorylation of Met in 786-O and MDA-MB-231 cell lines treated with mTOR inhibitors that have different mechanisms of action: everolimus, an allosteric mTORC1 inhibitor that acts through FKBP12 binding; PKI-587, a dual PI3K-mTOR kinase inhibitor; and OSI-027, a potent and selective inhibitor of mTOR complexes (mTORC) 1 and 2 [17]. Phospho-p70S6K served as marker of activity for all mTOR inhibitors. Compared with everolimus, neither PKI-587 nor OSI-027 inhibited Met phosphorylation at doses that reduced phospho-p70S6K (Supplementary Figure S1A).

**Table 1:** Effect of everolimus on Met TK catalytic activity

	D1228H	D1228N	F1200I	M1250T	Wild-type	Y1230A	Y1230C	Y1230D	Y1230H
Compound	IC50 (μM)	IC50 (μM)	IC50 (μM)	IC50 (μM)	IC50 (μM)	IC50 (μM)	IC50 (μM)	IC50 (μM)	IC50 (μM)
**everolimus**	>10	>10	>10	>10	>10	>10	>10	>10	>10
**PHA665752**	4.37	6.35	0.734	0.108	0.0185	4.22	3.57	7.92	1.88

Compound concentrations in the assay from 0.3 nM to 10 μM, semi-long step, singlicate measurement.

Ranking of IC_50_ values:

**Table:** 

	IC50 (μM) above 10
	IC50 (μM) between 10 and 1
	IC50 (μM) between 1 and 0.1
	IC50 (μM) below 0.1

To verify that Met phosphorylation is not directly related to mTOR inhibition, we used small interference RNA (siRNA) to silence different components of the mTOR complexes, namely, mTOR, Raptor and Rictor, in 786-O cells. mTOR partecipates in both mTORC1 and mTORC2 complexes, while Raptor and Rictor are part of only mTORC1 and mTORC2, respectively [5]. As expected, p70S6K phosphorylation was inhibited by both mTOR and Raptor but not by Rictor siRNAs. Neither silencing of mTOR, Raptor or Rictor affected Met phosphorylation (Supplementary Figure S1B). These data exclude that everolimus-dependent inhibition of Met is related to mTOR blockade.

### Everolimus inhibits Met phosphorylation via FKBP12

**Figure 2:**
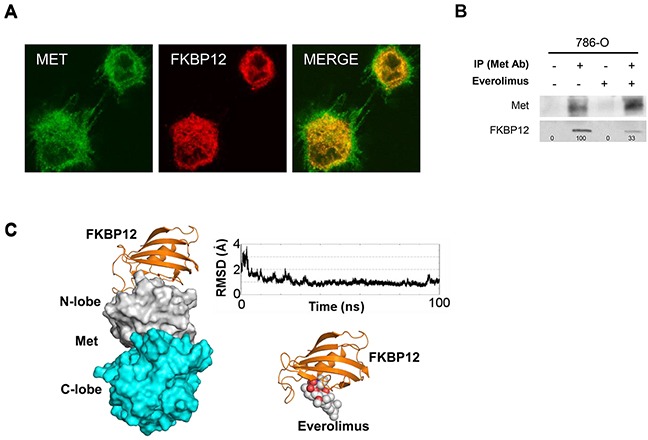
Everolimus inhibits phospho-Met phosphorylation via FKBP12 **A.** Colocalization analysis performed by immunofluorescence analysis: 786-O cells were grown on glass cover slips for 24 hours, then double-stained with anti-Met receptor and anti-FKBP12 primary antibodies and incubated with the appropriate rhodamine- or fluorescein-tagged goat anti-mouse or anti-rabbit antibody. **B.** Immunoprecipitation (IP) assay: 786-O cells, cultured in complete medium or treated for 24 hours with everolimus (0.5 μM), were immunoprecipitated using anti-Met antibody (Met Ab) and blotted with anti-Met and anti-FKBP12 antibodies. The same samples with normal IgG served as negative control. **C.** Computational analysis. Left: Calculated FKBP12/Met complex. FKBP12 is shown as orange ribbons while Met is shown as white and cyan surface for the N- and C-lobe, respectively. Top right: RMSD calculated for the FKBP12 backbone atoms along the 100-ns molecular dynamics simulation with respect to the FKBP12/Met average complex calculated over the 100 ns MD simulation. Bottom right: Everolimus/FKBP12 complex. The protein is shown as orange ribbons and the ligand as white and red spheres. The complex was calculated starting from the published X-ray rapamycin/FKBP12 complex.

As everolimus exerts its mechanism of action by binding to FKBP12, we asked whether everolimus reduces phospho-Met via FKBP12. We first studied the intracellular relationship between FKBP12 and Met in 786-O cells by immunofluorescence analysis. As expected, Met was prevalently localized on the cell membrane, whereas FKBP12 was widely distributed in various intracellular compartments and in juxtamembrane regions. Notably, as shown in the merge staining, Met and FKBP12 partially co-localized (Figure 2A).

To investigate in greater detail the potential functional/structural relationship between FKBP12 and Met, we immunoprecipitated total cell lysates from everolimus-treated and -untreated 786-O cells with the anti-Met antibody and blotted with the anti-FKBP12 antibody. As shown in Figure 2B, Met co-immunoprecipitated with FKBP12, which is consistent with the partial co-localization observed in immunofluorescence analysis. Moreover, the amount of FKBP12 co-immunoprecipitated with Met was lower in everolimus-treated 786-O cells (Figure 2B). We also carried out a computational study to evaluate the experimentally demonstrated Met-FKBP12 interaction at molecular level. To date, no structural information is available about the Met/FKBP12 complex. FKBP12 has been solved in complex with two kinases, type I TGF-β (TGFβI) [18] and type I activin receptor (Alk2) [19]: in both cases FKBP12 interacts with the N-ter region of the N-lobe of the kinase domain. Analysis of the whole eukaryotic phylogenetic tree for the kinase protein domain revealed that TGFβI, Alk2 and Met are in close branches (Supplementary Figure S2A). Docking analysis suggested that Met interacts with FKBP12 through its N-lobe domain (Supplementary Figure S2B). In addition, molecular dynamics simulation of the FKBP12/Met complex demonstrated stable specific interactions between the two proteins (Supplementary Figure S2C). Interestingly, a comparison between the everolimus/FKBP12 complex and FKBP12/Met suggests that Met and everolimus compete for the same FKBP12 region (Figure 2C).

### Everolimus does not inhibit Met phosphorylation in everolimus-resistant cancer cell lines

**Figure 3:**
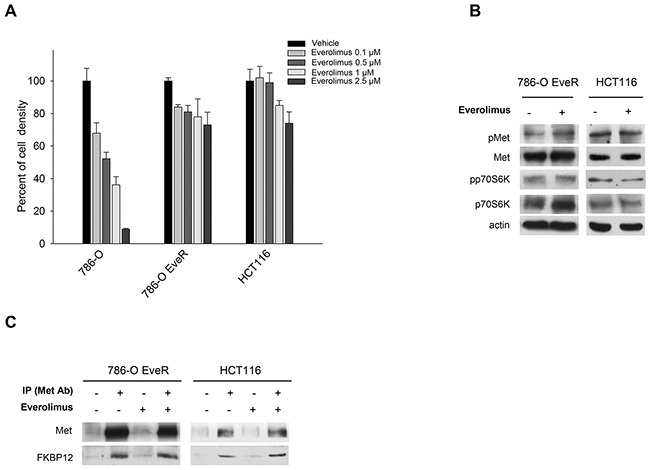
Everolimus does not inhibit Met phosphorylation in human everolimus resistant cancer cell lines **A.** Percent of cell density of 786-O, 786-O EveR and HCT116 cells treated for 72 hours with everolimus (0.1–2.5 μM), as measured by MTT assay. Data represent the mean (±SD) of three independent experiments, each performed in triplicate. Bars, SDs. **B.** Western blot analysis of protein expression in 786-O EveR and HCT116 cells treated for 24 hours with everolimus (0.5 μM). The relative optical density of phospho-protein levels normalized to total protein levels is shown. **C.** Immunoprecipitation assay: 786-O EveR and HCT116 cells, cultured in complete medium and treated for 24 hours with everolimus (0.5 μM), were immunoprecipitated using anti-Met antibody and blotted with anti-Met and an anti-FKBP12 antibodies. The same samples with normal IgG served as negative control.

To explore how Met activation affects sensitivity to everolimus, we generated renal cell carcinoma 786-O EveR (everolimus-resistant) cells with acquired resistance to everolimus from the 786-O parental cell line (Supplementary Methods). Moreover, HCT116 colon cancer cells have been used as a model of intrinsic resistance [13]. 786-O EveR and HCT116 cells are resistant to everolimus (Figure 3A); the concentration of everolimus causing 50% reduction of cell density was ≥ 5 μM (data not shown). Linear regression analysis showed that differences between the slopes were statistically significant (786-O EveR *vs* 786-O, *P* <0.01; HCT116 *vs* 786-O, *P* <0.05). Neither Met inhibition nor p70S6K phosphorylation occurred in the two cell lines after everolimus treatment (Figure 3B). Importantly, FKBP12 binds Met, even in a condition of everolimus resistance, as shown by immunoprecipitation assay (Figure 3C). Unlike data obtained in everolimus-sensitive models, the amount of FKBP12 co-immunoprecipitated with Met was not reduced in everolimus-treated resistant cells (Figure 3C).

### Met inhibition restores sensitivity to everolimus in resistant cell lines

**Figure 4:**
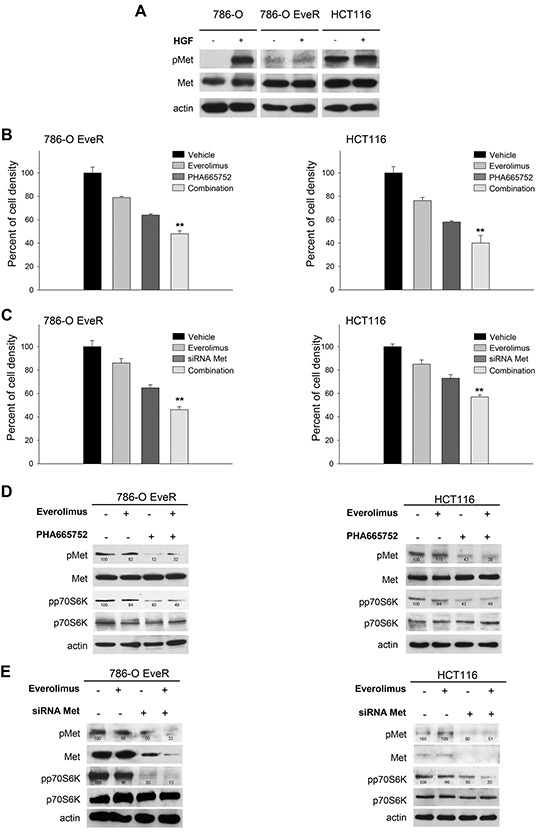
Met inhibition restores sensitivity to everolimus in resistant cell lines **A.** Western blot analysis of Met and phospho-Met in 786-O, 786-O EveR and HCT116 cells. Cell were cultured without serum for 24 hours or treated with HGF 50 ng/ml for 60 minutes. The relative optical density of phospho-protein levels normalized to total protein levels is shown. **B.** Percent of cell density of 786-O EveR and HCT116 cells treated for 72 hours with everolimus (1 μM), PHA665752 (1 μM) and combinations of both drugs as measured by MTT assay. **, 2-sided *P* < 0.01, combination versus PHA665752 alone. Data represent the mean (±SD) of three independent experiments, each performed in triplicate. Bars, SDs. **C.** Percent of cell density of 786-O EveR and HCT116 cells treated for 72 hours with everolimus (1 μM), siRNA Met (50 nM) and combinations of both as measured by MTT assay. **, 2-sided *P* < 0.01, combination versus Met siRNA alone. Data represent the mean (±SD) of three independent experiments, each performed in triplicate. Bars, SDs. **D.** Western blot analysis of protein expression in 786-O EveR and HCT116 cells treated for 24 hours with everolimus (1 μM), PHA665752 (1 μM) and combination of both drugs. The relative optical density of phospho-protein levels normalized to total protein levels is shown. **E.** Western blot analysis of protein expression in 786-O EveR and HCT116 cells treated for 24 hours with everolimus (1 μM), siRNA Met (50 nM) and combination of both. The relative optical density of phospho-protein levels normalized to total protein levels is shown.

To investigate the role of Met in the context of everolimus resistance, we analyzed Met phosphorylation levels in the absence and presence of HGF in 786-O, 786-O EveR and HCT116 cells. In everolimus-resistant cell lines high levels of phosphorylated/activated Met were detectable in the absence of HGF; conversely, in everolimus-sensitive cell lines phospho-Met is not detectable without HGF stimulation (Figure 4A). No difference in HGF expression levels were observed between sensitive and resistant cells (data not shown). To better define the contribution of Met to everolimus resistance, we evaluated the effect of Met inhibitor PHA665752 and Met silencing on everolimus-resistant cells. As shown in Figure 4B and 4C, combination of everolimus with both PHA665752 or Met siRNA significantly inhibits cell growth of everolimus-resistant cells, *P* <0.01 (Supplementary Table S2). As expected, in western blot analysis everolimus did not affect the phosphorylation of Met or p70S6K. Either PHA665752 or Met siRNA, alone and in combination with everolimus, reduced the phosphorylation of Met and p70S6K (Figure 4D, 4E).

To evaluate whether Met activation leads to everolimus resistance, we transiently transfected T47D cells (physiologically not expressing Met receptor) with vectors harboring wt Met or constitutively active Met mutants (Y1235D and M1268T). Transfection efficiency was confirmed by Western blot analysis (Supplementary Figure S3A). Compared with T47D-empty vector, cells with wt Met, and Y1235D and M1268T mutants were resistant to everolimus; the drug concentration causing 50% reduction of cell density was > 0.5 μM. In these cells, PHA665752 significantly restored sensitivity to everolimus, *P* <0.01 (Supplementary Figure S3B and Supplementary Table S3).

### Met inhibition cooperates with everolimus in nude mice subcutaneously xenografted with resistant cells

To investigate the simultaneous blockade of Met and mTOR in *in vivo* models of everolimus resistance, we first performed subcutaneous transplantation of resistant HCT116 cells in nude mice. The subcutaneous injection was used to evaluate tumor growth and mice survival.

**Figure 5:**
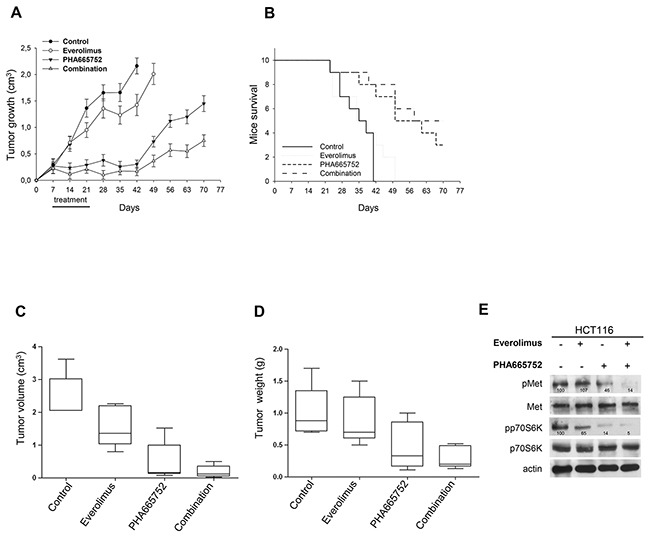
Inhibition of Met cooperate with everolimus in *in vivo* models of everolimus resistance **A.** Graph (box plots) shows tumor volumes of HCT116 orthotopic murine cancer models randomized (10/group) to receive everolimus, PHA665752 or their combination, as described in the Methods section. The horizontal line is a median (50th percentile) of the measured volumes, the top and bottom of the boxes represent 25th and 95th percentiles, respectively, and whiskers indicate the range from the largest to smallest observed data points within 1.5 interquartile range presented by the box. Comparison of tumor volume was statistically significant for both combination *vs* control and combination *vs* everolimus (*P* < 0.005), but not for combination vs PHA665752 (*P* = 0.326). **B.** Graph (box plots) shows tumor weights of HCT116 orthotopic murine cancer models randomized (10/group) to receive everolimus, PHA665752 or their combination, as described in the Methods section. The horizontal line is a median (50th percentile) of the measured volumes, the top and bottom of the boxes represent 25th and 95th percentiles, respectively, and whiskers indicate the range from the largest to smallest observed data points within 1.5 interquartile range presented by the box. Comparison of tumor weight was statistically significant for both combination *vs* control and combination *vs* everolimus (*P* < 0.05), but not for combination vs PHA665752 (*P* = 0.371). **C.** Western blot analysis was performed on total lysates from tumor specimens of mice sacrificed. Tumors derived from each treatment group were pooled during lysis to obtain a single specimen. **D.** Graph show tumor volume of HCT116 subcutaneous murine cancer models randomized (10/group) to receive everolimus, PHA665752 or their combination, as described in the Methods section. The one-way ANOVA test was used to compare tumor sizes among treatment groups at the median survival time of the control group (37 days). The results are statistically significant for the combination *vs* control (*P* < 0.0001), everolimus (*P* < 0.0001), or PHA665752 (*P* < 0.05). Bars, SDs. **E.** Graph show survival of HCT116 subcutaneous murine cancer models randomized (10/group) to receive everolimus, PHA665752 or their combination, as described in the Methods section. Median survival differences were statistically significant for the combination *vs* control (*P* = 0.0005) and combination *vs* everolimus (*P* = 0.0022), but not for combination *vs* PHA665752 (*P* = 0.446, log-rank test).

Balb/c nude mice subcutaneously xenografted with everolimus-resistant HCT116 cells were randomly assigned to one of four groups (10 mice for each group) to receive one of the following treatments: everolimus 5 mg/kg *per os*, five times a week for 2 weeks; PHA665752 20 mg/kg intravenous (i.v.), five times a week for 2 weeks or the combination of these agents. Untreated mice reached the maximum tumor size allowed on day 42, 6 weeks after cell injection. At this time point, everolimus and PHA665752 alone inhibited tumor growth by 35% and 85%, respectively, while the combination inhibited tumor growth by 90% (Figure 5A). PHA665752, alone and even more in combination with everolimus, exerted a strong and persistent antitumor activity until the end of the experiment (30% and 65% of tumor growth inhibition, respectively). Comparison of tumor sizes, evaluated by the one-way ANOVA test, was statistically significant for combination *vs* control, combination *vs* everolimus (both, *P* < 0.001), and combination *vs* PHA665752 (*P* < 0.05) at median survival of control group (Figure 5A). Consistently, 50% of mice treated with the everolimus/PHA665752 combination were alive at the end of the experiment. Median survival in the combination-treated mice was significantly longer than in control mice and in everolimus-treated mice, but not in mice exposed to PHA665752 (Figure 5B and Supplementary Table S4). Both everolimus and PHA665752 were well tolerated, and no significant loss of animal weight was observed in mice exposed to combined treatment. These data are consistent with the efficacy of PHA665752 in combination with rapamycin, previously demonstrated by Ma et al [20].

### Met inhibition cooperates with everolimus in nude mice orthotopically xenografted with resistant cells

To further investigate the simultaneous blockade of Met and mTOR in *in vivo* models of everolimus resistance, we also performed orthotopic transplantation of resistant HCT116 cells in nude mice. The orthotopic model was used to evaluate growth and invasion of tumor cells in their natural location. When orthotopic tumors, assessed with micro-ultrasonography, reached a mean volume of 0.6-0.7 cm^3^, mice were randomly divided into four groups (10 mice/group) to receive everolimus 5 mg/kg *per os*, five times a week for 2 weeks, PHA665752 20 mg/kg intravenous (i.v.), five times a week for 2 weeks or their combination. Untreated mice reached the maximum tumor size allowed, about 2 cm^3^, four weeks after tumor implantation. At this time point, mice were sacrificed, primary tumors excised and tumor volume/weight measured (Supplementary Methods). Only 5 mice/group survived, the other animals died from bowel obstruction during the experiment. As depicted in Figure 5C and Supplementary Figure S4, there were large tumors in the cecum and peritoneum of mice treated with vehicle and with everolimus. Treatment with PHA665752 greatly reduced tumor volume (Figure 5C) and tumor weight (Figure 5D); this effect was more pronounced with combination treatment. Comparison of tumor volume and tumor weight was statistically significant for combination *vs* control (*P* = 0.0001 and *P* = 0.008, respectively) and for combination *vs* everolimus (*P* = 0.0016 and *P* = 0.016, respectively), but not for combination vs PHA665752 (*P* = 0.326 and *P* = 0.371, respectively). Western blot analysis of tumors removed at the end of the experiment showed that the combination reduced the phosphorylation of both p70S6K and Met (Figure 5D).

## DISCUSSION

One of the great challenges of cancer research is to tailor therapy to each cancer patient. Consequently, the mechanisms of action of targeted therapies and the causes of limited therapeutic responses must be carefully assessed [21]. To date, everolimus, an orally available mTOR inhibitor approved for the treatment of advanced breast cancer, neuroendocrine tumors of pancreatic origin, and advanced renal cell carcinoma, has met multiple clinical needs in oncology [1]. The existence of negative feedbacks on RTKs sustained by p70S6K has been described for IGFR1 [22–27; 16] and platelet-derived growth factor receptor (PDGFR) [28]. Herein, we demonstrate that everolimus inhibits activation of the Met RTK in various everolimus-sensitive cancer cell lines. These results are consistent with previous data demonstrating Met dephosphorylation after everolimus treatment [29]. In our hands this effect is strictly related to interaction between FKBP12 and Met and not to the mTOR/p70S6K axis. The FKBP family includes immunophilin proteins endowed with prolyl isomerase activity [30] that interact with kinases and hormone receptors and thus probably play a relevant role in pathological processes as cancer [31]. FKBPs are implicated in cell growth and survival, in apoptotic signaling pathways, and moreover their expression was shown to differ between cancer tissues and non-tumor samples [32]. Various functions have been attributed to FKBPs: FKBP52 regulates steroid hormone receptors in breast and prostate cancer cells [33–35], FKBP51 regulates Akt [36] and NF-kB pathways [37], and FKBP65 is able to directly interact with cRAF-1 [38]. Interestingly, changes in intracellular FKBP12 levels could modulate EGFR autophosphorylation levels, which suggests that FKBP12 functions as an endogenous inhibitor of EGFR activation [8,39]. FKBP12 also acts as an interactor and a regulator of the type I serine/threonine kinase receptor of the TGF-beta superfamily [9,18,40,41].

We have identified a new role of FKBP12, namely, as a regulator of Met activation, which is supported by a phylogenetic rationale. Indeed, FKBP12 interacts with the N-lobe of the kinase domains of type I TGF-β and type I activin receptors (TGFβI and Alk2, respectively) that are phylogenetically closed to Met [40,19]. This suggests that, like TGFβI and Alk2, also Met should be able to make direct contact with FKBP12 through its N-lobe kinase domain. In this context, everolimus, by disrupting the FKBP12/Met complex, could facilitate Met inactivation. Resistance to everolimus prevents the dissociation of the FKBP12/Met complex, thus avoiding Met inactivation.

We suggest that increased Met activation could induce everolimus resistance. Little is known about factors predictive of response to everolimus, or about the mechanisms underlying everolimus resistance. Mutations in tuberous sclerosis complex 1 (TSC1) and 2 (TSC2), which encode negative regulators of the mTOR pathway, confer sensitivity to everolimus [42,43], while mutations in mTOR or FKBP12 induce resistance [44]. Also aberrant activation of the PI3K/Akt or Ras/MAPK pathways have been implicated in everolimus resistance, however, we are still far from fully understanding how everolimus resistance is established, how to treat everolimus refractory patients and how to identify everolimus sensitive patients [14]. In our hands, Met inhibition by both siRNA and PHA665752 produced a reduction in the activation/phosphorylation of p70S6K. In some cases, the combination of everolimus with Met inhibition did not potentiate this effect. Therefore, we hypothesize that activation of p70S6K could be one of the mechanisms through which activation of Met contribute to everolimus resistance.

In conclusion, our experimental data have potentially relevant clinical implications. First, we assign a new role to FKBP12, as a regulator of Met activation. Second, we suggest that everolimus should be considered not only an allosteric mTOR inhibitor, but also a Met inhibitor. Therefore, Met expression/activation could serve as a predictive biomarker of sensitivity to everolimus. Even if our results did not show synergism of action between everolimus and PHA665752, we found that Met inhibitor is effective in condition of everolimus resistance. Therefore, we suggest Met inhibition as an effective strategy to be used, secondarily to everolimus,in cancer patients affected by tumors with intrinsic or acquired resistance to everolimus.

## MATERIALS AND METHODS

### Compounds

Everolimus (RAD001), PHA665752, PKI-587 and OSI-027 were purchased from Selleck Chemicals (Germany). Human recombinant HGF was purchased from R&D Systems (Italy).

### Cell cultures

Human renal cell carcinoma (786-O, ACHN), breast (MDA-MB-231, MDA-MB-361, T47D), and colorectal (HCT116) cancer cell lines were obtained between 2010 and 2013 from the American Type Culture Collection (ATCC). All cells were maintained according to the manufacturer's protocol. Human non small cell lung cancer cell lines (PC-9 and NCI-H1975) were provided by Dr F. Morgillo (Second University of Naples) in 2012. 786-O EveR (everolimus-resistant) cells were generated according to a validated protocol of *in vivo*/*in vitro* selection after chronic exposure to the drug, as described [45].

### Cell lines authentications

Short tandem repeat (STR) profiles of cell lines were obtained using nine highly polymorphic STR loci plus amelogenin (Cell IDTM System, Promega). The amplified fragments were analyzed with the ABI PRISM 3100 Genetic Analyzer. Data analysis was performed by GeneMapper® software, version 4.0. Cell lines authentications was performed by IRCCS Azienda Ospedaliera Universitaria San Martino – Istituto Nazionale per la Ricerca sul Cancro (Genova, Italy). The cells were last tested between april and august 2015.

### Cell density assay

Cells (10^4^ cells/well) were grown in 24-well plates and exposed to increasing doses of everolimus and PHA665752, alone or in combination. The percentage of cell density was determined using the 3-(4,5-dimethylthiazol-2-yl)-2,5-diphenyltetrazolium bromide (MTT) assay according to the manufacturer's instructions (Sigma-Aldrich, Milan, Italy). The dose-response curves for each agent alone and in combination were determined at a fixed ratio based on the drug concentration causing 50% inhibition of cell proliferation.

### Transfection of small interfering RNA (siRNA)

Transfection of siRNAs (200 pmol) targeting mTOR, Rictor, Raptor, FKBP12 and Met was carried out according to the manufacturer's instructions (Dharmacon Inc., Lafayette, CO, USA). We used a scrambled siRNA as negative control. To evaluate target silencing, total protein was extracted 24 and 48 hours after transfection, and examined by western blot.

### Western blot and immunoprecipitation analyses

Total protein extracts obtained from cell cultures or tumor specimens were resolved by 4-15% SDS-PAGE and probed with anti-human, polyclonal pMet Y1349, polyclonal Met, monoclonal pp70S6K T412 and p70S6K (Merck-Millipore Darmstadt, Germany), monoclonal actin (Sigma-Aldrich, Milan, Italy), monoclonal FKBP12, polyclonal mTOR, Raptor, and Rictor. Co-immunoprecipitation analyses were performed with anti-Met; membranes were blotted with anti-FKBP12. The total lysate from 786-O, 786-O EveR and HCT116 cells served as positive control. Immunoreactive proteins were visualized by enhanced chemiluminescence (Pierce, Rockford, IL, USA). Densitometry was performed with Image J software (NIH, Bethesda, MD, USA).

### Fluorescence microscopy-confocal immunofluorescence

786-O cells (4 × 10^4^, seeded on sterile coverslips placed in 24-multiwell plates) were fixed with 4% paraformaldehyde solution and permeabilized with 0.2% triton x-100. They were then incubated for 1 hour at RT with monoclonal antibodies against FKBP12 (Santa Cruz-SC mouse, Santa Cruz, CA, USA) and polyclonal antibodies against Met (Cell Signaling, Beverly, MA, USA). Lastly, they were fluorescently labeled with the following secondary antibodies: Cy2-AffiniPure Donkey Anti-Rabbit IgG and Cy3-AffiniPure Donkey Anti-Mouse IgG (LiStarFish, Milan, Italy). Slides were mounted with glycerol 50% in PBS and imaged using a Zeiss LSM 510 meta confocal microscope equipped with an oil immersion plan apochromat 63x objective 1.4 NA.

### Subcutaneous and orthotopic murine colorectal cancer models

We subcutaneously xenografted everolimus-resistant HCT116 cells into 50 four- to six-week-old female BALB/c athymic nu+/nu+ (nude) mice (Charles River Laboratories, Milan, Italy). Forty animals were used to carry out the subcutaneous colorectal cancer model experiment; when tumors reached a mean volume of 1 cm^3^, 10 animals were euthanized, tumors were divided into 2-mm-sized pieces and microsurgically implanted in the cecum of 40 Balb/C nude mice for the orthotopic experiment. See Supplementary Methods for further details about the surgical procedure and the treatment schedule.

### Statistical analysis

The Student's *t* test was used to evaluate the statistical significance of the *in vitro* results. The statistical significance of differences in tumor growth was determined by one-way ANOVA and Dunnett's multiple comparison post-test, and that of differences in survival by a log-rank test [46]. The linear regression test was used to evaluate the statistical significance of the *in vitro* results of everolimus-resistant cells versus sensitive cells (Graph-Pad version 5). All reported *P* values were two-sided. Analyses were performed with the BMDP New System statistical package version 1.0 for Microsoft Windows (BMDP Statistical Software, Los Angeles, CA).

## SUPPLEMENTARY METHODS, FIGURES AND TABLES



## References

[1] Lebwohl D, Anak O, Sahmoud T, Klimovsky J, Elmroth I, Haas T, Posluszny J, Saletan S, Berg W (2013). Development of everolimus, a novel oral mTOR inhibitor, across a spectrum of diseases. Ann NY Acad Sci.

[2] Baselga J, Campone M, Piccart M, Burris HA, Rugo HS, Sahmoud T, Noguchi S, Gnant M, Pritchard KI, Lebrun F, Beck JT, Ito Y, Yardley D (2012). Everolimus in postmenopausal hormone-receptor-positive advanced breast cancer. N Engl J Med.

[3] Yardley DA, Noguchi S, Pritchard KI, Burris HA, Baselga J, Gnant M, Hortobagyi GN, Campone M, Pistilli B, Piccart M, Melichar B, Petrakova K, Arena FP (2013). Everolimus plus exemestane in postmenopausal patients with HR(+) breast cancer: BOLERO-2 final progression-free survival analysis. Adv Ther.

[4] Piccart M, Hortobagyi GN, Campone M, Pritchard KI, Lebrun F, Ito Y, Noguchi S, Perez A, Rugo HS, Deleu I, Burris HA, Provencher L, Neven P (2014). Everolimus plus exemestane for hormone-receptor-positive, human epidermal growth factor receptor-2-negative advanced breast cancer: overall survival results from BOLERO-2. Ann Oncol.

[5] Yang H, Rudge DG, Koos JD, Vaidialingam B, Yang HJ, Pavletich NP (2013). mTOR kinase structure, mechanism and regulation. Nature.

[6] Cameron AM, Steiner JP, Roskams AJ, Ali SM, Ronnett GV, Snyder SH (1995). Calcineurin associated with the inositol 1,4,5-trisphosphate receptor-FKBP12 complex modulates Ca2+ flux. Cell.

[7] Ahearn IM, Tsai FD, Court H, Zhou M, Jennings BC, Ahmed M, Fehrenbacher N, Linder ME, Philips MR (2011). FKBP12 binds to acylated H-ras and promotes depalmitoylation. Mol Cell.

[8] Mathea S, Li S, Schierhorn A, Jahreis G, Schiene-Fischer C (2011). Suppression of EGFR autophosphorylation by FKBP12. Biochemistry.

[9] Wang T, Donahoe PK (2004). The immunophilin FKBP12: a molecular guardian of the TGF-beta family type I receptors. Front Biosci.

[10] Motzer RJ, Escudier B, Oudard S, Hutson TE, Porta C, Bracarda S, Grünwald V, Thompson JA, Figlin RA, Hollaender N, Urbanowitz G, Berg WJ, Kay A (2008). Efficacy of everolimus in advanced renal cell carcinoma: a double-blind, randomised, placebo-controlled phase III trial. Lancet.

[11] Yao JC, Shah MH, Ito T, Bohas CL, Wolin EM, Van Cutsem E, Hobday TJ, Okusaka T, Capdevila J, de Vries EG, Tomassetti P, Pavel ME, Hoosen S (2011). RAD001 in Advanced Neuroendocrine Tumors, Third Trial (RADIANT-3) Study Group. Everolimus for advanced pancreatic neuroendocrine tumors. N Engl J Med.

[12] Ortolani S, Ciccarese C, Cingarlini S, Tortora G, Massari F (2015). Suppression of mTOR pathway in solid tumors: lessons learned from clinical experience in renal cell carcinoma and neuroendocrine tumors and new perspectives. Future Oncol.

[13] Di Nicolantonio F, Arena S, Tabernero J, Grosso S, Molinari F, Macarulla T, Russo M, Cancelliere C, Zecchin D, Mazzucchelli L, Sasazuki T, Shirasawa S, Geuna M (2010). Deregulation of the PI3K and KRAS signaling pathways in human cancer cells determines their response to everolimus. J Clin Invest.

[14] Carew JS, Kelly KR, Nawrocki ST (2011). Mechanisms of mTOR inhibitor resistance in cancer therapy. Target Oncol.

[15] Corso S, Giordano S (2013). Cell-autonomous and non-cell-autonomous mechanisms of HGF/MET-driven resistance to targeted therapies: from basic research to a clinical perspective. Cancer Discov.

[16] Buck E, Eyzaguirre A, Rosenfeld-Franklin M, Thomson S, Mulvihill M, Barr S, Brown E, O'Connor M, Yao Y, Pachter J, Miglarese M, Epstein D, Iwata KK (2008). Feedback mechanisms promote cooperativity for small molecule inhibitors of epidermal and insulin-like growth factor receptors. Cancer Res.

[17] Markman B, Dienstmann R, Tabernero J (2010). Targeting the PI3K/Akt/mTOR pathway--beyond rapalogs. Oncotarget.

[18] Huse M, Chen YG, Massagué J, Kuriyan J (1999). Crystal Structure of the Cytoplasmic Domain of the Type I TGF Beta Receptor in Complex with FKBP12. Cell.

[19] Chaikuad A, Alfano I, Kerr G, Sanvitale CE, Boergermann JH, Triffitt JT, von Delft F, Knapp S, Knaus P, Bullock AN (2012). Structure of the Bone Morphogenetic Protein Receptor ALK2 and Implications for Fibrodysplasia Ossificans Progressiva. J Biol Chem.

[20] Ma PC, Schaefer E, Christensen JG, Salgia R (2005). A selective small molecule c-MET Inhibitor, PHA665752, cooperates with rapamycin. Clin Cancer Res.

[21] Ramos P, Bentires-Alj M (2015). Mechanism-based cancer therapy: resistance to therapy, therapy for resistance. Oncogene.

[22] Harrington LS, Findlay GM, Gray A, Tolkacheva T, Wigfield S, Rebholz H, Barnett J, Leslie NR, Cheng S, Shepherd PR, Gout I, Downes CP, Lamb RF (2004). The TSC1-2 tumor suppressor controls insulin-PI3K signaling via regulation of IRS proteins. J Cell Biol.

[23] Hartley D, Cooper GM (2002). Role of mTOR in the degradation of IRS-1: regulation of PP2A activity. J Cell Biochem.

[24] Haruta T, Uno T, Kawahara J, Takano A, Egawa K, Sharma PM, Olefsky JM, Kobayashi M (2000). A rapamycin-sensitive pathway down-regulates insulin signaling via phosphorylation and proteasomal degradation of insulin receptor substrate-1. Mol Endocrinol.

[25] Takano A, Usui I, Haruta T, Kawahara J, Uno T, Iwata M, Kobayashi M (2001). Mammalian target of rapamycin pathway regulates insulin signaling via subcellular redistribution of insulin receptor substrate 1 and integrates nutritional signals and metabolic signals of insulin. Mol Cell Biol.

[26] Shah OJ, Wang Z, Hunter T (2004). Inappropriate activation of the TSC/Rheb/mTOR/S6K cassette induces IRS1/2 depletion, insulin resistance, and cell survival deficiencies. Curr Biol.

[27] Tamburini J, Chapuis N, Bardet V, Park S, Sujobert P, Willems L, Ifrah N, Dreyfus F, Mayeux P, Lacombe C, Bouscary D (2008). Mammalian target of rapamycin (mTOR) inhibition activates phosphatidylinositol 3-kinase/Akt by up-regulating insulin-like growth factor-1 receptor signaling in acute myeloid leukemia: rationale for therapeutic inhibition of both pathways. Blood.

[28] Zhang H, Bajraszewski N, Wu E, Wang H, Moseman AP, Dabora SL, Griffin JD, Kwiatkowski DJ (2007). PDGFRs are critical for PI3K/Akt activation and negatively regulated by mTOR. J Clin Invest.

[29] Lin CI, Whang EE, Donner DB, Du J, Lorch J, He F, Jiang X, Price BD, Moore FD, Ruan DT (2010). Autophagy induction with RAD001 enhances chemosensitivity and radiosensitivity through Met inhibition in papillary thyroid cancer. Mol Cancer Res.

[30] Ivery MT (2000). Immunophilins: switched on protein binding domains?. Med Res Rev.

[31] Solassol J, Mange A, Maudelonde T (2011). FKBP family proteins as promising new biomarkers for cancer. Curr Opin Pharmacol.

[32] Romano S, D'Angelillo A, Romano MF (2015). Pleiotropic roles in cancer biology for multifaceted proteins FKBPs. Biochim Biophys Acta.

[33] Cheung-Flynn J, Prapapanich V, Cox M.B, Riggs DL, Suarez-Quian C, Smith DF (2005). Physiological role for the cochaperone FKBP52 in androgen receptor signaling. Mol Endocrinol.

[34] Ward BK, Mark PJ, Ingram DM, Minchin RF, Ratajczak T (1999). Expression of the estrogen receptor-associated immunophilins, cyclophilin 40 and FKBP52, in breast cancer. Breast Cancer Res Treat.

[35] Periyasamy S, Warrier M, Tillekeratne MP, Shou W, Sanchez ER (2007). The immunophilin ligands cyclosporin A and FK506 suppress prostate cancer cell growth by androgen receptor-dependent and-independent mechanisms. Endocrinology.

[36] Pei H, Li L, Fridley BL, Jenkins GD, Kalari KR, Lingle W, Petersen G, Lou Z, Wang L (2009). FKBP51 affects cancer cell response to chemotherapy by negatively regulating Akt. Cancer Cell.

[37] Bouwmeester T, Bauch A, Ruffner H, Angrand PO, Bergamini G, Croughton K, Cruciat C, Eberhard D, Gagneur J, Ghidelli S, Hopf C, Huhse B, Mangano R (2004). A physical and functional map of the human TNF-alpha/NF-kappa B signal transduction pathway. Nat Cell Biol.

[38] Coss MC, Stephens RM, Morrison DK, Winterstein D, Smith LM, Simek SL (1998). The immunophilin FKBP65 forms an association with the serine/threonine kinase c-Raf-1. Cell Growth Differ.

[39] Lopez-Ilasaca M, Schiene C, Küllertz G, Tradler T, Fischer G, Wetzker R (1998). Effects of FK506-binding protein 12 and FK506 on autophosphorylation of epidermal growth factor receptor. J Biol Chem.

[40] Chen YG, Liu F, Massague J (1997). Mechanism of TGFbeta receptor inhibition by FKBP12. EMBO J.

[41] Okadome T, Oeda E, Saitoh M, Ichijo H, Moses HL, Miyazono K, Kawabata M (1996). Characterization of the interaction of FKBP12 with the transforming growth factor-beta type I receptor in vivo. J Biol Chem.

[42] Iyer G, Hanrahan AJ, Milowsky MI, Al-Ahmadie H, Scott SN, Janakiraman M, Pirun M, Sander C, Socci ND, Ostrovnaya I, Viale A, Heguy A, Peng L (2012). Genome sequencing identifies a basis for everolimus sensitivity. Science.

[43] Wagle N, Grabiner BC, Van Allen EM, Amin-Mansour A, Taylor-Weiner A, Rosenberg M, Gray N, Barletta JA, Guo Y, Swanson SJ, Ruan DT, Hanna GJ, Haddad RI (2014). Response and acquired resistance to everolimus in anaplastic thyroid cancer. N Engl J Med.

[44] Fruman DA, Wood MA, Gjertson CK, Katz HR, Burakoff SJ, Bierer BE (1995). Fk506 binding protein 12 mediates sensitivity to both FK506 and Rapamycin in murine mast cells. Eur J Immunol.

[45] Rosa R, Monteleone F, Zambrano N, Bianco R (2014). In vitro and in vivo models for analysis of resistance to anticancer molecular therapies. Curr Med Chem.

[46] Rosa R, Melisi D, Damiano V, Bianco R, Garofalo S, Gelardi T, Agrawal S, Di Nicolantonio F, Scarpa A, Bardelli A, Tortora G (2011). Toll-like receptor 9 agonist IMO cooperates with cetuximab in K-ras mutant colorectal and pancreatic cancers. Clin Cancer Res.

