# Supplementary material for: Everolimus induces Met inactivation by disrupting the FKBP12/Met complex
Source: Oncotarget. 2016 May 19;7(26):40073–84. doi: 10.18632/oncotarget.9484 (PMC5129993; doi:10.18632/oncotarget.9484)
Supplement: Supplementary file 1 [file oncotarget-07-40073-s001.pdf]

Everolimus induces Met inactivation by disrupting the FKBP12/Met complex

SUPPLEMENTARY METHODS

Generation of resistant cell lines

786-Ocells were injected subcutaneously in nude mice. When tumors reached about 0.5 cm^3, mice were treated with everolimus 2.5 mg/kg *per os*, five times a week for 8 weeks. Tumors that progressed under everolimus treatment were excised, and resistant cells, 786-O EveR, were maintained *in vitro* and tested for everolimus sensitivity.

In vitro Met kinase inhibition assay

The Met kinase inhibition by everolimus was analyzed with a Met kinase mutant profile screening service by ProQinase (ProQinase GmbH, Freiburg, Germany). Briefly, compounds (everolimus *vs* PHA665752) were tested at 10 different concentrations (standard range: $3x10^{-10}$M–$1x10^{-5}$M; semilog dilutions) against human recombinant wt Met and nine Met mutant protein kinases, and IC_{50} values were calculated. IC_{50} values of Met reference inhibitor (PHA665752) were determined side-by-side. All assays were performed at the corresponding apparent ATP Km of each protein kinase using the radiometric ^{33}PanQinase Assay™.

Computational analysis

More than 60 X-ray crystal structures for Met kinase are available in PDB. Among them, we selected, as reference X-ray, the kinase that has: (*i*) a resolution below 2.00 Å; (*ii*) the largest portion of the kinase domain solved; and (*iii*) an active conformation. The resulting selected Met structure is in its unbound dually-phosphorylated state (pdb code: 3Q6U) [Rickert KW, *J Biol Chem.* 2011]. Regarding FKBP12, we selected the 1FKR NMR solution structure [Michnick SW, *Science.* 1991]. Indeed, FKBP12 undergoes great conformational changes at the 80 loop level [Mustafi SM, *Biochem J.* 2014]. Among the 20 frames available in the NMR solution structure, we selected the one with the greatest extension [distance (Å) between Ans43 and Gly89 Cα] at the 80 loop level (frame 13). To generate the Met/FKBP12 complex, we searched the whole PDB database for X-ray crystal structures of kinases/FKBP12 complexes. Based on results obtained, protein-protein docking studies were carried out only on the N-ter region of the Met N-lobe domain, using the HADDOCK 2.2 software web portal [de Vries SJ, *Proteins.* 2007; Dominguez Z, *J Am Chem Soc.* 2003]. To assess the stability and, in turn, the reliability of the generated Met/FKBP12 complex, a 100-ns long molecular dynamics (MD) simulation was conducted. The selected complex was refined by using the Protein Preparation tool available in Maestro9.1. This tool enables one to consider the protonation state of the amino acid residues and to geometrically refine the complex. Missing loops and side chains were refined using Prime. Subsequently, both proteins were capped with ACE and NME residues at their N-ter and C-ter residues, respectively. The resulting complex was embedded in an explicit water box model (TIP3P) and parametrized using the amber *ff*99SB*ildn* force filed (final size and atom count: 105 x 68 x 59 Å and 37.456, respectively) [http://ambermd.org/]. The system obtained was equilibrated using NAMD2.8 [Phillips JC, *J Comput Chem.* 2005], and the final MD simulation was conducted applying a protocol similar to that previously described [Capelli AM, *J Med Chem.* 2013]. The final Met/FKBP12 complex was obtained from the cluster analysis performed on all the MD trajectories. The complex was aligned on the Cα atoms of Met, while the backbone atoms Root-mean-square deviation (RMSD) of FKBP12 was measured and used to cluster the trajectories. The cluster analysis was performed using ptraj and the average-linkage algorithm with a cut-off value of 1 Å. Finally, the representative structure of the most populated cluster was choosen as the final Met/FKBP12 complex. Ptraj turned out an average structure and a representative structure (the frame closest to the average structure in terms of RMSD) for each cluster.

Cell transduction with lentiviral vectors

Cells were transduced using third-generation Lentiviral vectors with the polypurine tract sequence [Follenzi A, *Nat Genet.* 2000]. As transfer vector, we used the pRRL.sin.PPT.hCMV.pre, where the full-length *MET* cDNA (4284 bp) was subcloned as a *NotI-XhoI* fragment. Mutations were introduced in the human *MET* cDNA using a PCR-based technique, as described elsewhere [Bardelli A, *PNAS.* 1998.]. The tyrosine kinase domain of *MET* cDNA containing each mutation was substituted in the above transfer vector as *SpeI-SwaI* insert for Y1253D and M1268T mutants. We used the pRRL.sin.PPT.hCMV.GFP.pre vector as control. Serial dilutions of freshly harvested conditioned medium were used to infect 10^5 T47D cells in a six-well plate in the presence of Polybrene (8 μg/ml).

Subcutaneous murine colorectal cancer model

Five weeks old BALB/cAnNCrlBR athymic (nu+/nu+) mice (Charles River Laboratories, Milan, Italy) maintained in accordance with institutional guidelines of the University of Naples Federico II Animal Care Committee and in accordance to the Declaration of Helsinki were injected subcutaneously with HCT116

cells (107 cells/mice) resuspended in 200 μL of Matrigel (Collaborative Biomedical Products, Bedford, MA, USA). Fourteen days after tumor cells injection, tumor-bearing mice were randomly assigned (n = 10 per group) to receive the following: everolimus 5 mg/kg per os, five times a week for 2 weeks; PHA665752 20 mg/kg intravenous (i.v.), five times a week for 2 weeks [Christensen JG, Cancer Res. 2003] or the combination of these agents. Tumor diameter was assessed with a vernier caliper, and tumor volume (cm3) was measured with the formula p/6 x larger diameter x (smaller diameter)2. Mice were sacrificed when the tumor reached the size of about 2 cm3, the maximum size allowed by the Ethics Committee.

Orthotopic murine colorectal cancer model

For the orthotopic implantation in the mice cecum,the recipient animals were anesthetized with 2,2,2-tribomoethanol 97% TBE, Avertin (Sigma-Aldrich, St. Louis, MO, USA). TBE solution was prepared fresh daily by mixing 0.625 g of 97% crystalline TBE powder with 25 ml sterile 0.9% saline, and injected intraperitoneally at 0.01 ml/g body mass (250 mg/kg). The abdomen was prepared with betadine solution and the surgical site was isolated in a sterile fashion. A laparotomy of 0.5 cm was conducted; the cecum was exteriorized and isolated using pre-cut, sterile gauze. A warm saline solution was used to keep the cecum wet. Subsequently, the cecum wall was slightly damaged with a 30G needle and a tumor fragment from HCT116 subcutaneous tumors was sutured to the mesenteric border of the cecum wall using 6.0 nylon surgical sutures. The cecum was then placed into the abdominal cavity and the abdominal wound was sutured using a 7.0 Ethicon absorbable stitches (Ethicon Inc., Somerville, NJ). Seven days after implantation, mice were randomly assigned to one of four groups (10 mice for each group) to receive one of the following treatments: everolimus 5 mg/kg *per os*, five times a week for 2 weeks; PHA665752 20 mg/kg intravenous (i.v.), five times a week for 2 weeks [Christensen JG, *Cancer Res.* 2003] or the combination of these agents. Tumor diameter was assessed with ultrasonography (VEVO, Visualsonics Inc., Toronto, Canada) before treatment and once a week during follow-up. Body weights were monitored daily. Mice were sacrificed when, four weeks after tumor implantation, tumor volume of untreated mice, calculated with ultrasonography, reached the size of about 2 cm^3, which is the maximum size allowed by the Ethics Committee. Primary tumors in the cecum were excised and weighed. The final tumor was measured with a caliper and the volume was calculated with the following formula: $\pi/6$ x larger diameter x (smaller diameter)^2.

REFERENCES

1. Bardelli A, Longati P, Gramaglia D, Basilico C, Tamagnone L, Giordano S, Ballinari D, Michieli P, Comoglio PM. Uncoupling signal transducers from oncogenic MET mutants abrogates cell transformation and inhibits invasive growth. Proc Natl Acad Sci U S A. 1998; 95: 14379-14383.
2. Capelli AM, Bruno A, Entrena Guadix A, Costantino G. Unbinding pathways from the glucocorticoid receptor shed light on the reduced sensitivity of glucocorticoid ligands to a naturally occurring, clinically relevant mutant receptor. J Med Chem. 2013; 56: 7003-7014.
3. Christensen JG, Schreck R, Burrows J, Kuruganti P, Chan E, Le P, Chen J, Wang X, Ruslim L, Blake R, Lipson KE, Ramphal J, Do S, Cui JJ, Cherrington JM, Mendel DB. A selective small molecule inhibitor of c-Met kinase inhibits c-Met-dependent phenotypes in vitro and exhibits cytoreductive antitumor activity in vivo. Cancer Res. 2003; 63: 7345-7355.
4. de Vries SJ, van Dijk AD, Krzeminski M, van Dijk M, Thureau A, Hsu V, Wassenaar T, Bonvin AM HADDOCK versus HADDOCK: new features and performance of HADDOCK2.0 on the CAPRI targets. Proteins. 2007; 69: 726-733.
5. Dominguez Z, Khuong TA, Dang H, Sanrame CN, Nuñez JE, Garcia-Garibay MA. Molecular compasses and gyroscopes with polar rotors: synthesis and characterization of crystalline forms. J Am Chem Soc. 2003; 125: 8827-8837.
6. Follenzi A, Ailles LE, Bakovic S, Geuna M, Naldini L. Gene transfer by lentiviral vectors is limited by nuclear translocation and rescued by HIV-1 pol sequences. Nat Genet. 2000; 25: 217-222.
7. Michnick SW, Rosen MK, Wandless TJ, Karplus M, Schreiber SL. Solution structure of FKBP, a rotamase enzyme and receptor for FK506 and rapamycin. Science. 1991; 252: 836-839.
8. Mustafi SM, LeMaster DM, Hernández G. Differential conformational dynamics in the closely homologous FK506-binding domains of FKBP51 and FKBP52. Biochem J. 2014; 461: 115-123.
9. Phillips JC, Braun R, Wang W, Gumbart J, Tajkhorshid E, Villa E, Chipot C, Skeel RD, Kalé L, Schulten K. Scalable molecular dynamics with NAMD. J Comput Chem. 2005; 26: 1781-1802.
10. Rickert KW, Patel SB, Allison TJ, Byrne NJ, Darke PL, Ford RE, Guerin DJ, Hall DL, Kornienko M, Lu J, Munshi SK, Reid JC, Shipman JM, Stanton EF, Wilson KJ, Young JR, Soisson SM, Lumb KJ. Structural basis for selective small molecule kinase inhibition of activated c-Met. J Biol Chem. 2011; 286: 11218-11225.

SUPPLEMENTARY FIGURES AND TABLES

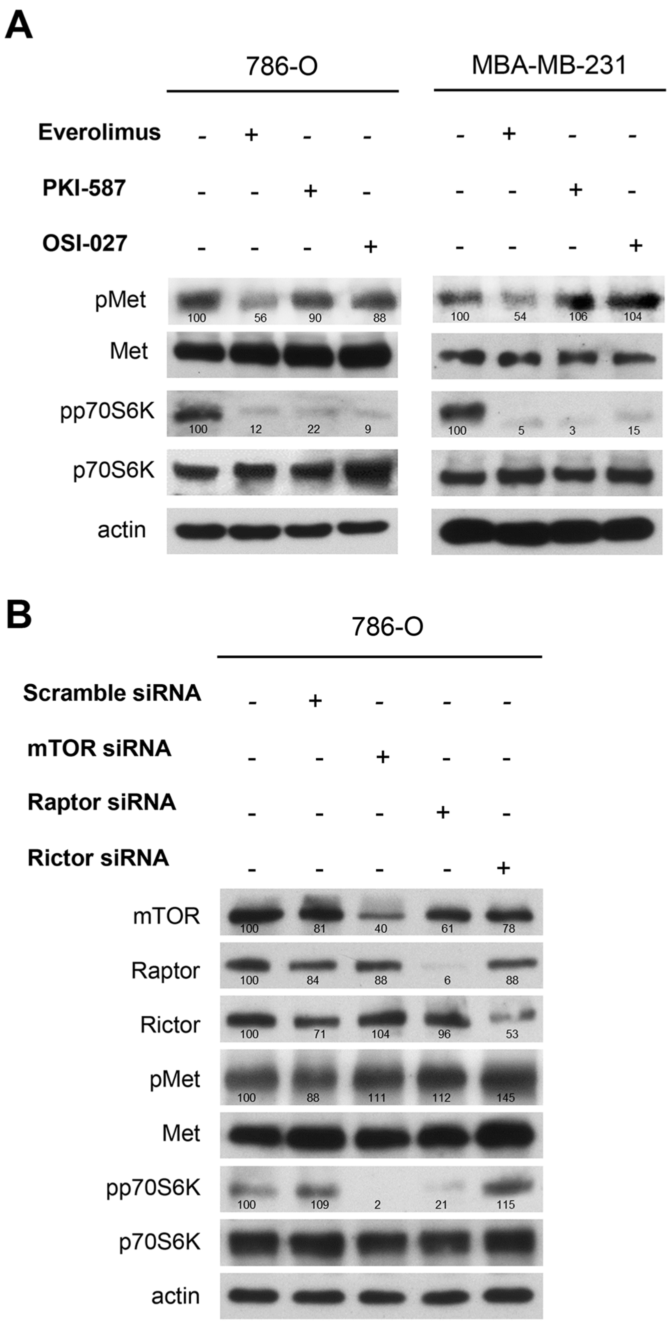

Supplementary Figure S1: Met phosphorylation is not reduced after mTOR inhibition.1 A. Western blot analysis of protein expression in 786-O cells and MDA-MB-231cells treated for 24 hours with everolimus (0.5 µM), PKI-587 (0.5 µM) or OSI-027 (0.5 µM). The relative optical density of phospho-protein levels normalized to total protein levels is shown. **B.** Western blot analysis of protein expression in 786-O cells treated for 48 hours with scrambled, mTOR, Raptor, and Rictor siRNAs. The relative optical density of phospho-protein levels normalized to total protein levels is shown.

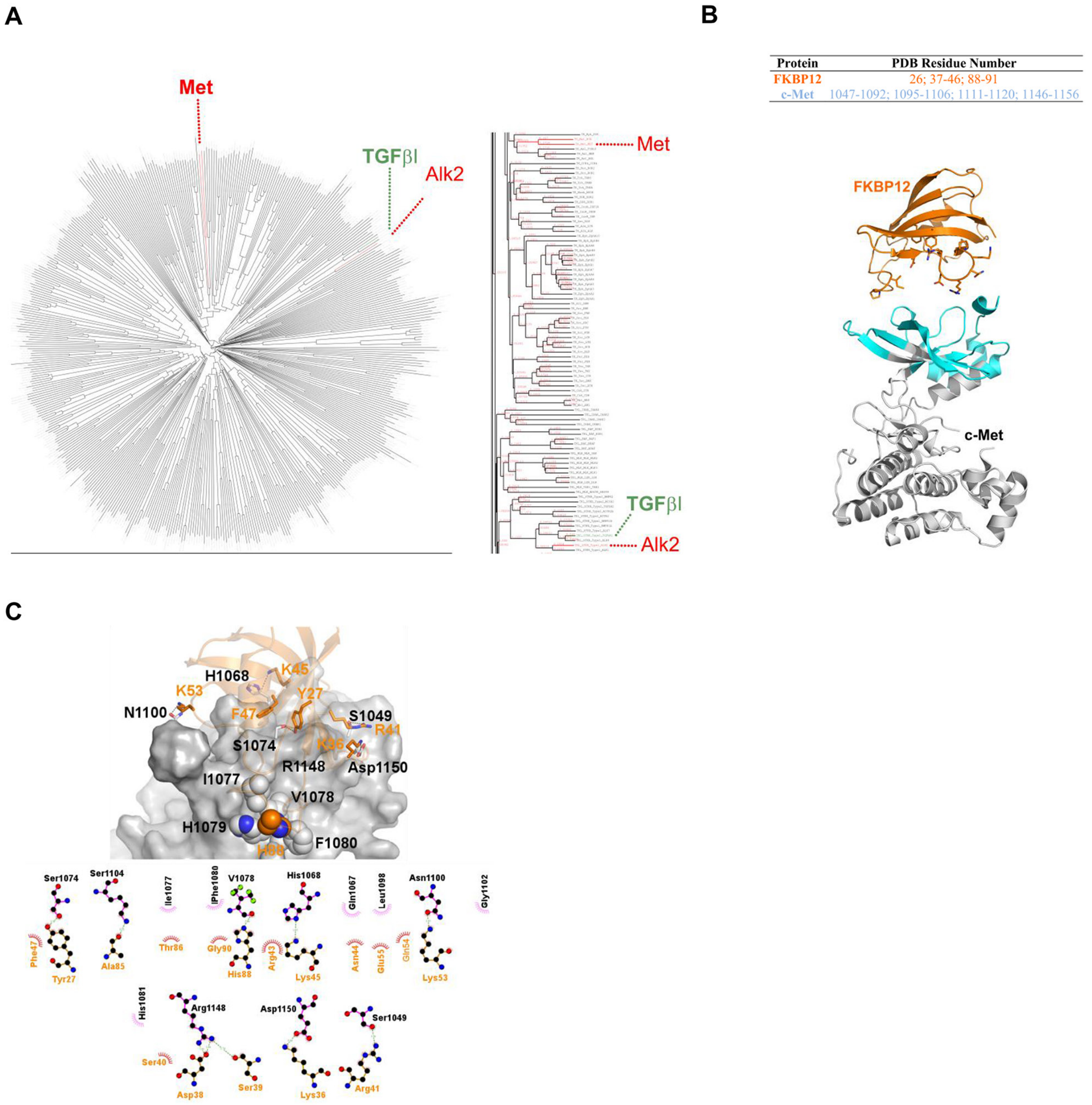

Supplementary Figure S2: FKBP12 interacts with Met. A. Phylogenetic tree of the whole eukaryotic kinome. The phylogenetic tree was obtained from the kinase.com “Genomics, evolution and function of protein kinases” project, at http://kinase.com/web/current/human/phylogeny. The circular tree and the phylogram were generated with TreeDyn. The kinases of interest are indicated. **B.** Region considered for the docking studies. FKBP12 is depicted as orange cartoon and sticks, while Met as white ribbons. Cyan cartoons depict the region of Met considered for the docking studies. The table shows the residue numbers (full length numeration) corresponding to the region considered for the docking studies. **C.** Top: Three-dimensional representation of the key interaction established by FKBP12 and Met. FKBP12 is depicted as orange ribbons and sticks, Met as white surface sticks and spheres. Bottom: Schematic plot of the most stable interactions established by FKBP12 (orange labeled residues) and Met (black labeled residues).

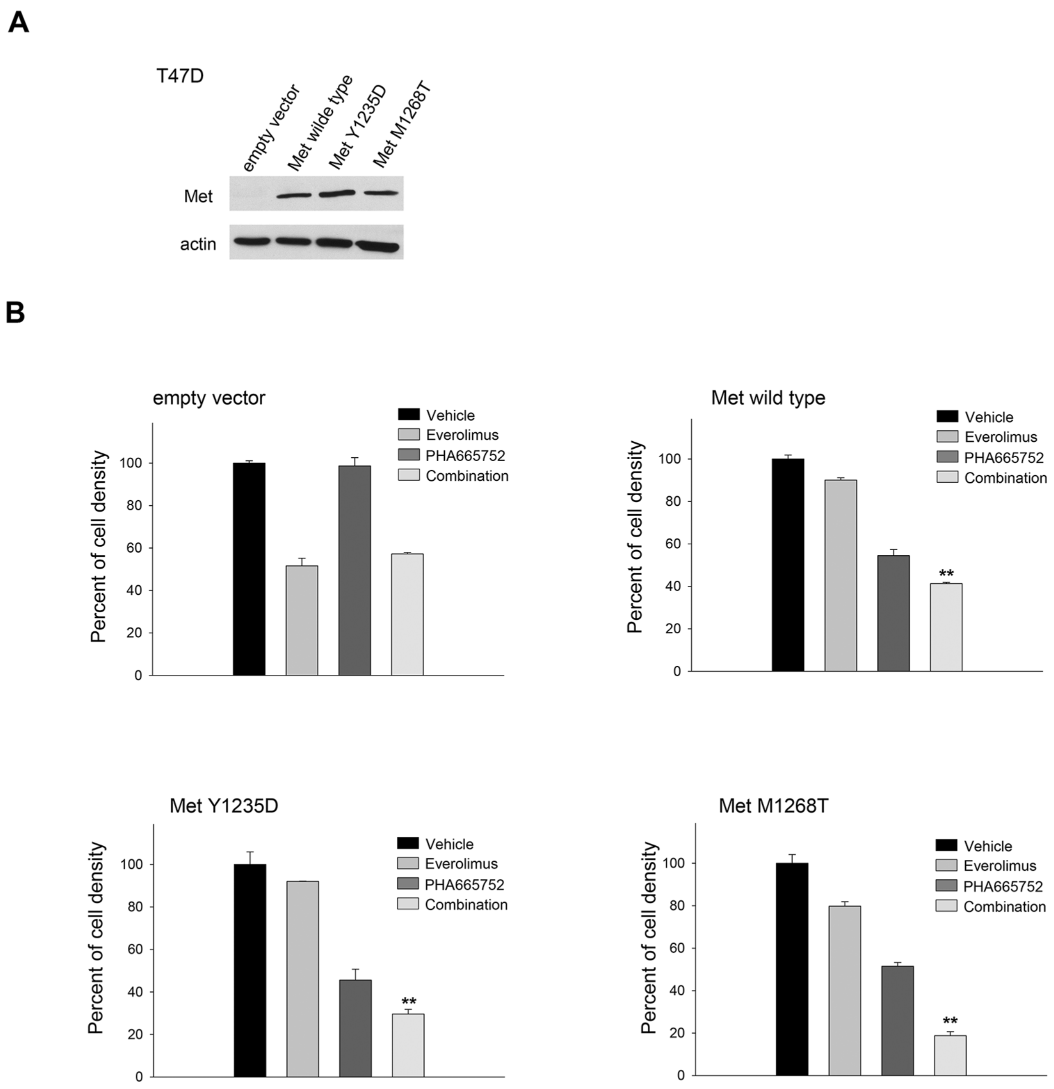

Supplementary Figure S3: Met constitutive activation correlates with everolimus resistance. A. Western blot analysis of Met in T47D cells transduced with lentiviral empty vector versus lentiviral vector harbouring Met wt, Met with Y1235D mutation and Met with M1268T mutation. **B.** Percent of cell density of T47D transiently transduced with empty vector, with Met wild type, with the Met Y1235D and Met M1268T mutations and treated for 72 hours with everolimus (1 µM), with PHA665752 (1 µM) or with a combination of both as measured by MTT assay. **, 2-sided $P < 0.01$, combination versus PHA665752 alone. Data represent the mean (±SD) of three independent experiments, each performed in triplicate. Bars, SDs.

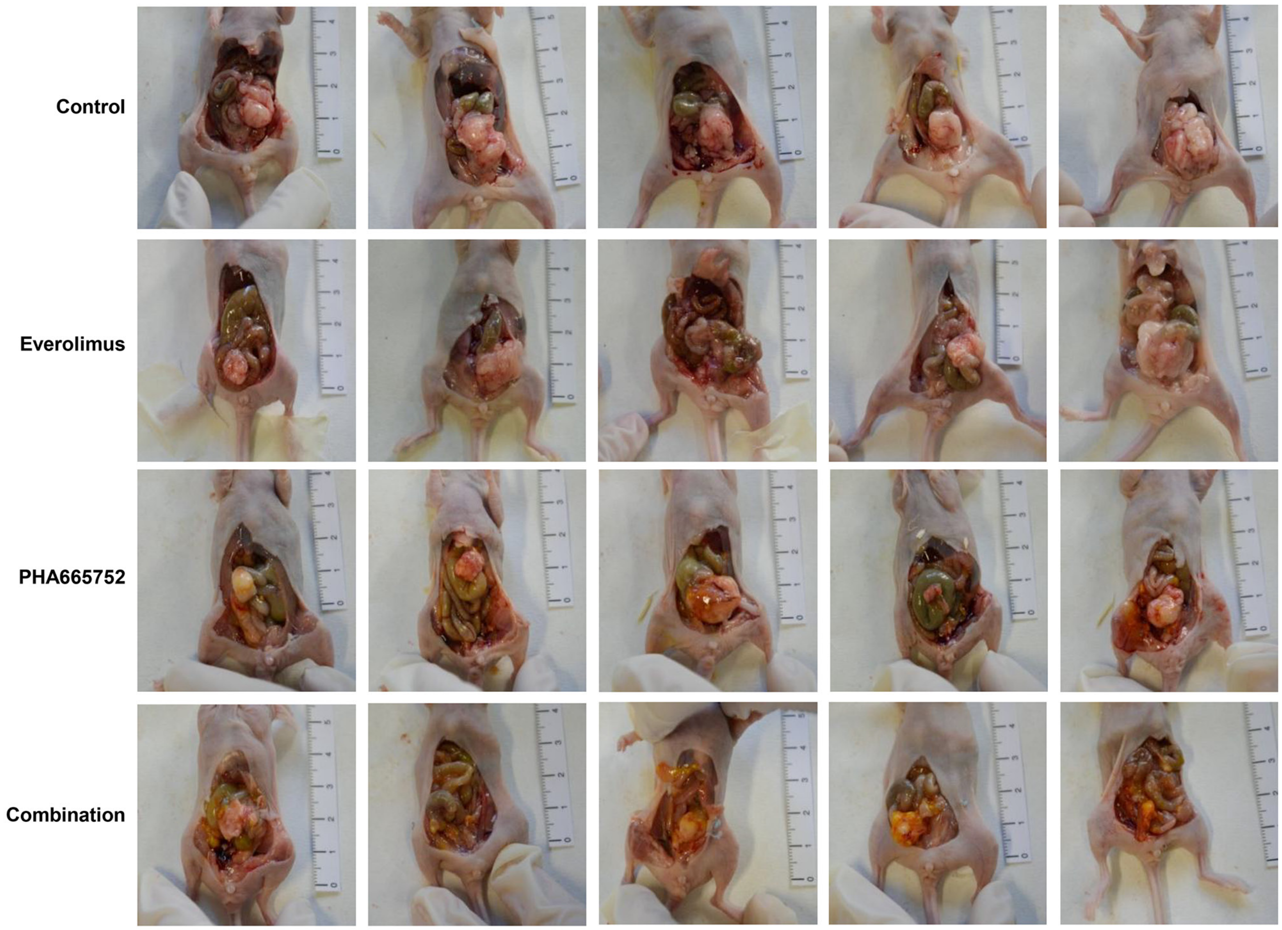

Supplementary Figure S4: A combination of Everolimus and PHA665752 inhibits growth of orthotopic HCT116 CRC xenografts. HCT116 cells were injected into the cecal wall of nude mice. Two weeks later, the mice were randomly assigned (10 mice each group) to receive: everolimus 5 mg/kg *per os*, five times a week for 2 weeks; PHA665752 20 mg/kg intravenous (i.v.), five times a week for 2 weeks or the combination of these agents. The treatment continued for 2 weeks, and 1 week later mice were killed and necropsied. Pictures of the animals included in the experiment (5 mice each group) are shown.

Supplementary Table S1: *P* values for cell density reduction by everolimus vs control in different human cancer cell lines, measured by MTT assay

Cell lines	0.1 µM	0.5 µM	1 µM	2.5 µM
**786-O**	< 0.001	< 0.001	< 0.001	< 0.001
**ACHN**	< 0.001	< 0.001	< 0.001	< 0.001
**MDA-MB-231**	< 0.001	< 0.001	< 0.001	< 0.001
**MDA-MB-361**	< 0.001	< 0.001	< 0.001	< 0.001
**PC-9**	< 0.001	< 0.001	< 0.001	< 0.001
**H1975**	< 0.001	< 0.001	< 0.001	< 0.001

Supplementary Table S2: *P* values for cell density reduction by everolimus plus PHA665752 vs PHA 665752 alone in 786-O EveR and HCT116 cell lines. *P* values for survival inhibition by everolimus plus siRNA Met vs siRNA Met in 786-O EveR and HCT116 cell lines

Cell line		p value
**786-O EveR**	combination vs PHA665752	< 0.001
**HCT116**	combination vs PHA665752	0.009
**786-O EveR**	combination vs siRNA Met	< 0.001
**HCT116**	combination vs siRNA Met	0.002

Supplementary Table S3: *P* values for cell density reduction by everolimus plus PHA 665752 vs PHA 665752 alone in T47D cells transfected with Met wild type, Met Y1253 or Met M1268T mutant variants

T47D		*P* value
**Met wild type**	combination *vs* PHA665752	0.002
**Met Y1253D**	combination *vs* PHA665752	0.007
**Met M1268T**	combination *vs* PHA665752	< 0.001

Supplementary Table S4: Statistical analysis of mice survival in the HCT116 subcutaneously xenografted model

	Median survival	Hazard ratio	95% CI	*P* value
**Combination vs control**	68 *vs* 37	0.1093	0.03147-0.3799	0.0005
**Combination vs everolimus**	68 *vs* 37.5	0.154	0.04644-0.5104	0.0022
**Combination vs PHA665752**	68 *vs* 57	0.6316	0.1937-2.059	0.446
